# Human Female Genital Tract Infection by the Obligate Intracellular Bacterium *Chlamydia trachomatis* Elicits Robust Type 2 Immunity

**DOI:** 10.1371/journal.pone.0058565

**Published:** 2013-03-13

**Authors:** Rodolfo D. Vicetti Miguel, Stephen A. K. Harvey, William A. LaFramboise, Seth D. Reighard, Dean B. Matthews, Thomas L. Cherpes

**Affiliations:** 1 Department of Pediatrics, University of Pittsburgh School of Medicine, Pittsburgh, Pennsylvania, United States of America; 2 Department of Ophthalmology, University of Pittsburgh School of Medicine, Pittsburgh, Pennsylvania, United States of America; 3 Department of Pathology, University of Pittsburgh School of Medicine, Pittsburgh, Pennsylvania, United States of America; Duke University Medical Center, United States Of America

## Abstract

While *Chlamydia trachomatis* infections are frequently asymptomatic, mechanisms that regulate host response to this intracellular Gram-negative bacterium remain undefined. This investigation thus used peripheral blood mononuclear cells and endometrial tissue from women with or without *Chlamydia* genital tract infection to better define this response. Initial genome-wide microarray analysis revealed highly elevated expression of matrix metalloproteinase 10 and other molecules characteristic of Type 2 immunity (e.g., fibrosis and wound repair) in *Chlamydia*-infected tissue. This result was corroborated in flow cytometry and immunohistochemistry studies that showed extant upper genital tract *Chlamydia* infection was associated with increased co-expression of CD200 receptor and CD206 (markers of alternative macrophage activation) by endometrial macrophages as well as increased expression of GATA-3 (the transcription factor regulating T_H_2 differentiation) by endometrial CD4^+^ T cells. Also among women with genital tract *Chlamydia* infection, peripheral CD3^+^ CD4^+^ and CD3^+^ CD4^-^ cells that proliferated in response to *ex vivo* stimulation with inactivated chlamydial antigen secreted significantly more interleukin (IL)-4 than tumor necrosis factor, interferon-γ, or IL-17; findings that repeated in T cells isolated from these same women 1 and 4 months after infection had been eradicated. Our results thus newly reveal that genital infection by an obligate intracellular bacterium induces polarization towards Type 2 immunity, including *Chlamydia*-specific T_H_2 development. Based on these findings, we now speculate that Type 2 immunity was selected by evolution as the host response to *C. trachomatis* in the human female genital tract to control infection and minimize immunopathological damage to vital reproductive structures.

## Introduction

*Chlamydia trachomatis* is an obligate intracellular Gram-negative bacterium that infects human ocular and genital epithelium. Ocular *C. trachomatis* infection causes trachoma, an important cause of preventable blindness whose earlier stages are often asymptomatic [1]. Typically, *C. trachomatis* genital tract infection is also asymptomatic, a feature enhancing its sexual transmission [2]. When untreated, female genital tract *Chlamydia* infection may cause Fallopian tube damage that increases the risk of ectopic pregnancy and infertility [3]. More often, however, even long-standing infection is cleared in the absence of overt genital tract damage, while advancing age is associated with increased resistance to infection [4], [5]. Such observations imply the formation of *Chlamydia*-specific protective immunity and the possibility of developing a prophylactic vaccine (provided better understanding of human host response to natural *C. trachomatis* genital tract infection is achieved).

In cogitation of a clinical picture signaling that *C. trachomatis* infection does not elicit the robust inflammation that drives differentiation of T_H_1 and T_H_17 immunity, our lab posited that Type 2 immunity (including T_H_2-type responses) represents the primary defense against *Chlamydia* in the human female genital tract [6]. This hypothesis opposed current dogma, developed in murine models of genital *Chlamydia muridarum* infection, which maintains that response to *C. trachomatis* in the human genital tract is similarly dominated by Type 1 immunity [7]. Providing context for the formation and validity of our alternative hypothesis, Type 2 immunity is induced by numerous microbes that establish chronic infection, creating tissue environments that dampen inflammation and promote wound healing [8]. Playing a pivotal role in this response are IL-4-secreting T_H_2 cells that stimulate macrophages to promote tissue repair (i.e., alternative macrophage activation) [9]. Although Type 2 immunity is established as an important defense against extracellular parasites, its role against intracellular parasites is not well explored. Offering preliminary, albeit indirect evidence for the formation of *Chlamydia*-specific Type 2 immunity, our lab detected only short-lived T_H_1 and negligible T_H_17 *Chlamydia*-specific immunity among women with documented history of *C. trachomatis* infection [10]. Because of these unremarkable *Chlamydia*-specific T_H_1 and T_H_17 responses, in the current study, peripheral blood mononuclear cells (PBMC) and endometrial tissue from women with or without genital *C. trachomatis* infection were used to determine if this intracellular bacterium is instead a more potent inducer of T_H_2 immunity. As posited, *C. trachomatis* infection of genital tissue stimulated robust Type 2 immunity, including T_H_2 differentiation, alternative macrophage activation, and increased expression of IL-24 and other molecules enhancing tissue repair. Of equal importance, we observed that secretion of IL-4, and not IFN-γ or IL-17, was the principal effector function of peripheral T cells responding to *ex vivo* stimulation with chlamydial antigen. Taken together, these results newly uncover exuberant Type 2 immunity elicited upon *C. trachomatis* infection of the human female genital tract.

## Results and Discussion

**Figure 1:**
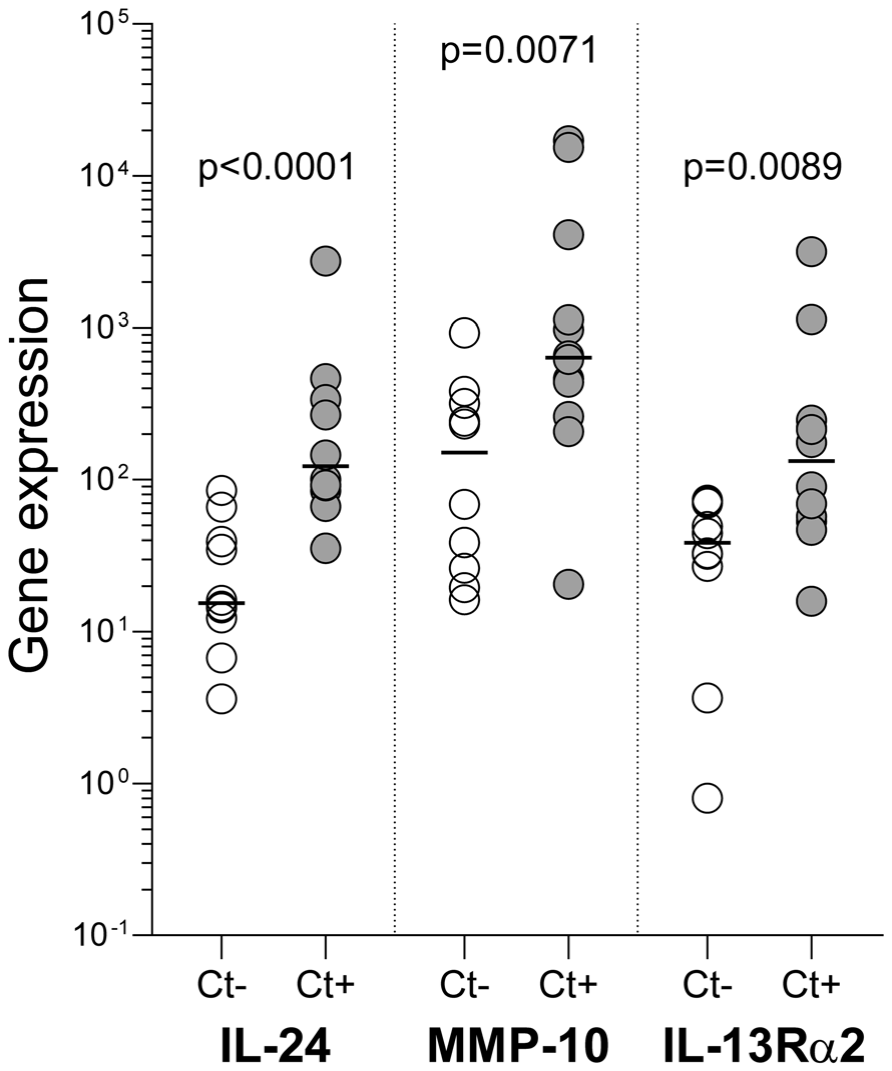
Genome-wide microarray analysis shows *C. trachomatis* elicits robust Type 2 immunity. Compared to expression in uninfected controls, endometrial tissue from women with existing endometrial *Chlamydia* infection displayed 15-fold, 13-fold, and 11-fold increases in the expression of MMP-10, IL-24, and IL-13Rα2, respectively. These genes, each with biological activity linked to Type 2 immunity, were 3 of the 4 most dramatically upregulated genes in *Chlamydia*-infected tissue. Significance of differences between groups was determined by use of Dunn’s test (see Methods section for further details regarding statistical considerations). Open circles indicate samples from uninfected controls (n = 10); gray circles indicate samples from women with existing endometrial *Chlamydia* infection (n = 12) (horizontal bars indicate median values for each group).

To begin our investigation of host response to *C. trachomatis* in the human female genital tract, microarrays that compared gene expression in uninfected and *Chlamydia*-infected endometrial tissue were performed. Initial analysis of this data showed that *Chlamydia* infection caused significant enrichment of canonical pathways associated with Type 2 immunity [11], including pathways involved in fibrosis and wound repair (Table 1). Moreover, 3 of the 4 genes most highly upregulated in *Chlamydia*-infected tissue, matrix metalloproteinase 10 (MMP10) (15-fold increase), IL-13α_2_ receptor (IL-13Rα_2_) (13-fold increase), and IL-24 (11-fold increase), regulate biological functions that are characteristic of Type 2 immunity (Figure 1 and Table 2). MMP-10, a metalloproteinase produced by T cells in response to IL-4, stimulates wound healing [12], [13]; while interactions between IL-13 to IL-13Rα2, also regulated by IL-4, promotes tissue repair by increasing production of transforming growth factor-β1 [14], [15]. Likewise, IL-24 secretion by monocytes and T_H_2 cells increases the activity of signaling pathways responsible for wound healing [16]-[18]. Endometrial *Chlamydia* infection also induced a 10-fold increase in MUC5AC, a mucin gene expressed at low levels in normal endometrial tissue but upregulated by IL-4 [19], [20], and a 9-fold increase in aquaporin 4, an integral membrane protein highly upregulated among individuals with asthma [21] (Table 2).

**Table 1:** Canonical pathways significantly enriched (P < 0.01) in endometrial tissue of women with endometrial *C. trachomatis* infection vs. endometrial tissue of women with no existing upper or lower genital tract infection.

Ingenuity Canonical Pathways	-log (p), i.e. 2 ≡ p < 0.01	# Genes up-regulated	# Genes down-regulated	# Genes in Pathway
Hepatic Fibrosis / Hepatic Stellate Cell Activation	6.03	22	9	82
B Cell Development	4.34	10	0	71
Primary Immunodeficiency Signaling	3.23	11	0	196
Communication between Innate and Adaptive Immune Cells	2.72	14	0	65
Hematopoiesis from Pluripotent Stem Cells	2.51	9	0	61
Role of Macrophages, Fibroblasts and Endothelial Cells in Rheumatoid Arthritis	2.20	25	11	248
Acute Myeloid Leukemia Signaling	2.19	8	6	120
TREM1 Signaling	2.18	10	1	51
Metabolism of Xenobiotics by Cytochrome P450	2.11	12	1	95
Glycosphingolipid Biosynthesis – Neolactoseries	2.06	6	0	67
Autoimmune Thyroid Disease Signaling	2.04	8	0	95
Systemic Lupus Erythematosus Signaling	2.00	21	3	50
Amyotrophic Lateral Sclerosis Signaling	1.90	12	3	42
MSP-RON Signaling Pathway	1.89	8	1	151
Crosstalk between Dendritic Cells and Natural Killer Cells	1.85	14	0	206
GM-CSF Signaling	1.83	8	3	92
Allograft Rejection Signaling	1.82	8	0	526
Graft-versus-Host Disease Signaling	1.82	8	0	239
Thyroid Cancer Signaling	1.75	4	4	128
eNOS Signaling	1.74	13	5	74
Arachidonic Acid Metabolism	1.73	13	1	207
Altered T Cell and B Cell Signaling in Rheumatoid Arthritis	1.67	12	0	63
G-Protein Coupled Receptor Signaling	1.65	45	6	28
Role of Osteoblasts, Osteoclasts and Chondrocytes in Rheumatoid Arthritis	1.65	17	10	109
PTEN Signaling	1.60	13	3	89
Role of PI3K/AKT Signaling in the Pathogenesis of Influenza	1.53	7	3	49
Dendritic Cell Maturation	1.48	16	5	142
Nur77 Signaling in T Lymphocytes	1.40	8	0	84
Glycosphingolipid Biosynthesis – Lactoseries	1.39	3	0	79
Natural Killer Cell Signaling	1.39	11	3	82
Small Cell Lung Cancer Signaling	1.37	9	2	71
Docosahexaenoic Acid (DHA) Signaling	1.35	5	2	196
Ovarian Cancer Signaling	1.34	10	7	65
VEGF Family Ligand-Receptor Interactions	1.33	7	4	61
Non-Small Cell Lung Cancer Signaling	1.31	7	3	248
Eicosanoid Signaling	1.30	22	9	120

**Table 2:** List of the 20 molecules (and corresponding fold change) that were identified by genome-wide microarray analysis as the most intensely upregulated by endometrial *C. trachomatis* infection.

Entrez Gene Name	Fold change
matrix metallopeptidase 10 (stromelysin 2)	15.19
interleukin 24	13.40
corneodesmosin	12.61
interleukin 13 receptor, alpha 2	11.30
hydroxycarboxylic acid receptor 3	10.00
tripartite motif containing 48	10.00
thyroglobulin	9.85
tumor necrosis factor receptor superfamily, member 11b	9.71
pecanex homolog (Drosophila)	9.68
mucin 5AC, oligomeric mucus/gel-forming	9.54
carcinoembryonic antigen-related cell adhesion molecule 7	9.32
bone morphogenetic protein 15	9.09
desmocollin 3	9.89
mucin 3B, cell surface associated	8.81
dopamine receptor D5	8.80
cutaneous T-cell lymphoma-associated antigen 1	8.66
recombination activating gene 1	8.56
aquaporin 4	8.56
killer cell immunoglobulin-like receptor, three domains, X1	8.48
uncharacterized LOC100507630	8.45

**Figure 2:**
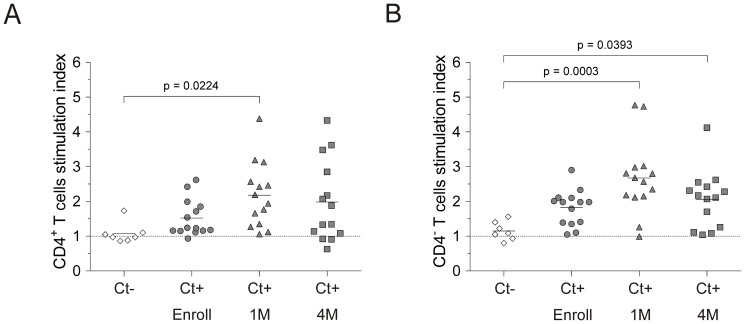
The ability of peripheral T cells from women with existing or treated *Chlamydia* infection to proliferate in response to stimulation with *C. trachomatis* elementary bodies (EB) decreased 4 months after antimicrobial administration. Peripheral blood mononuclear cells (PBMC) isolated from women at enrollment and at 1 and 4 m follow-up visits were cultured 96 h in presence of inactivated EB or media alone. Proliferation of (A) CD3^+^CD4^+^ and (B) CD3^+^CD4^-^ cells was assessed by flow cytometry using stimulation indexes calculated as described in Methods section. Stratification of *Chlamydia*-infected women by time since diagnosis and treatment of infection showed T cell proliferation was higher 1 month after treatment compared to enrollment, and that proliferative capacity diminished 4 months after treatment. Stimulation indexes of samples from *Chlamydia*-infected women (n = 14) at indicated visits were compared to those from women with no known history of infection (n = 7) using one-way ANOVA and Dunnett’s multiple comparison test (horizontal bars indicate means).

**Figure 3:**
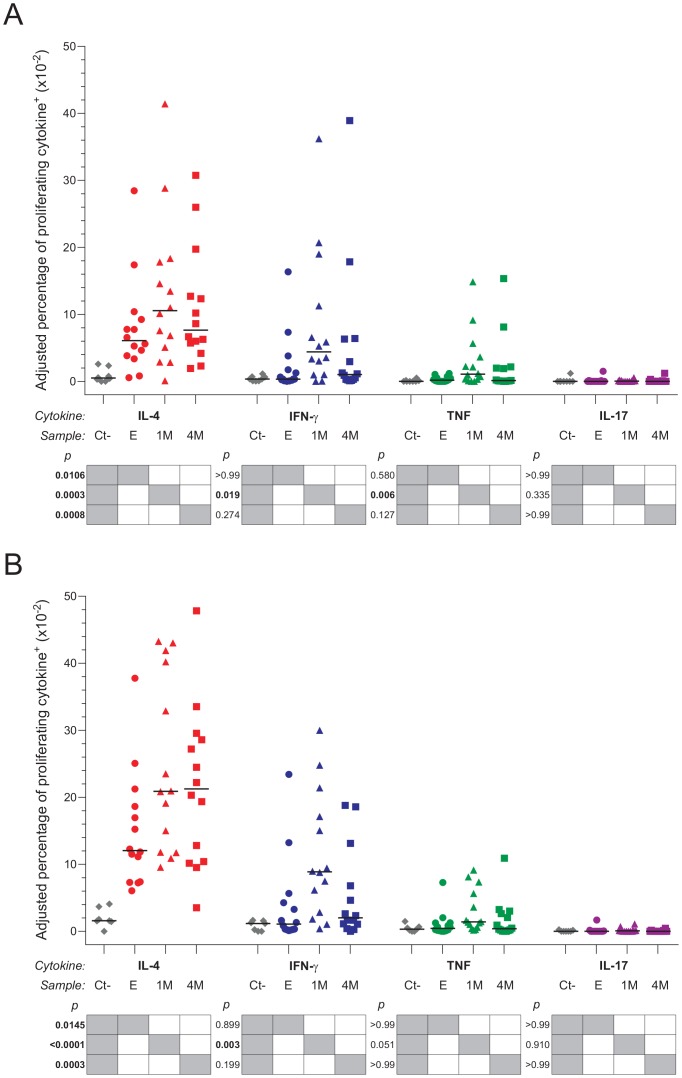
T_H_2-type immunity dominates host response to *C. trachomatis* infection. PBMC were isolated from women with no history of *Chlamydia* infection (n = 7) and women with an existing endocervical or endometrial *Chlamydia* infection (n = 14) at enrollment and again from the latter women 1 and 4 months after initiating an anti-chlamydial antimicrobial. Flow cytometric analysis of intracellular cytokine staining (ICS) allowed comparison of EB-stimulated (A) CD3^+^CD4^+^ and (B) CD3^+^CD4^-^ T cells that proliferated and produced IFN-γ, TNF, IL-4, or IL-17 (calculation described in Methods section). The adjusted percentages of cytokines that were produced in response to EB stimulation among uninfected and infected women were compared using Kruskal-Wallis’ test and Dunn’s post-hoc test (horizontal bars indicate medians). Grey boxes indicate pairs considered in the comparison for each p value displayed, and significant p values are indicated in bold characters.

As microarray analysis showed *C. trachomatis* promotes exuberant *in situ* differentiation of Type 2 immunity, we postulated this pathogen must also elicit T_H_2-type responses. To test this hypothesis, PBMC isolated from women with no *Chlamydia* infection history or women with existing (at enrollment) and then treated (at 1- and 4-month follow-up visits) endocervical or endometrial *Chlamydia* infection were used in intracellular cytokine staining (ICS) assays that used flow cytometry to delineate the effector function of T cells responding to stimulation with inactivated *C. trachomatis* elementary bodies (EB). As predicted, CD3^+^ cells in these assays from women with existing or treated *Chlamydia* infection proliferated in response to stimulation with inactivated EB (Figure S1). Interestingly, proliferation was more robust at the 1-month follow-up visit than at the enrollment or 4-month follow-up visits (Figure 2). Calculating the adjusted percentages of cytokines produced by peripheral CD3^+^ CD4^+^ or CD3^+^ CD4^-^ cells that proliferated in response to EB, we saw negligible production of IL-17 in samples from uninfected and *Chlamydia*-infected women at all study visits (Figure 3). Conversely, there was enhanced intracellular accumulation of IFN-γ and TNF by proliferating CD3^+^ CD4^+^ and CD3^+^ CD4^-^ cells from *Chlamydia*-infected women, but only in specimens collected at the 1-month follow-up visit (Figure 3). Interestingly, these results were congruent with our recently published cross-sectional study in which peripheral blood specimens obtained from *Chlamydia*-infected women 30-60 d after starting a *Chlamydia*-specific antimicrobial displayed a higher frequency of CD4^+^ cells producing IFN-γ in response to EB stimulation compared to specimens collected <30 d or > 60 d after starting therapy [10]. Even more interesting, in the current investigation we also found that intracellular IL-4 accumulation by proliferating CD3^+^ CD4^+^ and CD3^+^ CD4^-^ cells in PBMC samples from *Chlamydia*-infected women at enrollment, 1-month, and 4-month visits were all significantly higher than in uninfected controls (Figure 3 and Figure S2). This indicated that *Chlamydia*-specific T cells were preferentially polarized towards a T_H_2 profile, and together with our earlier publication, suggested that *Chlamydia*-specific T_H_1 immunity develops more slowly, is more transient, and is perhaps a less biologically relevant host response than *Chlamydia*-specific T_H_2 immunity.

**Figure 4:**
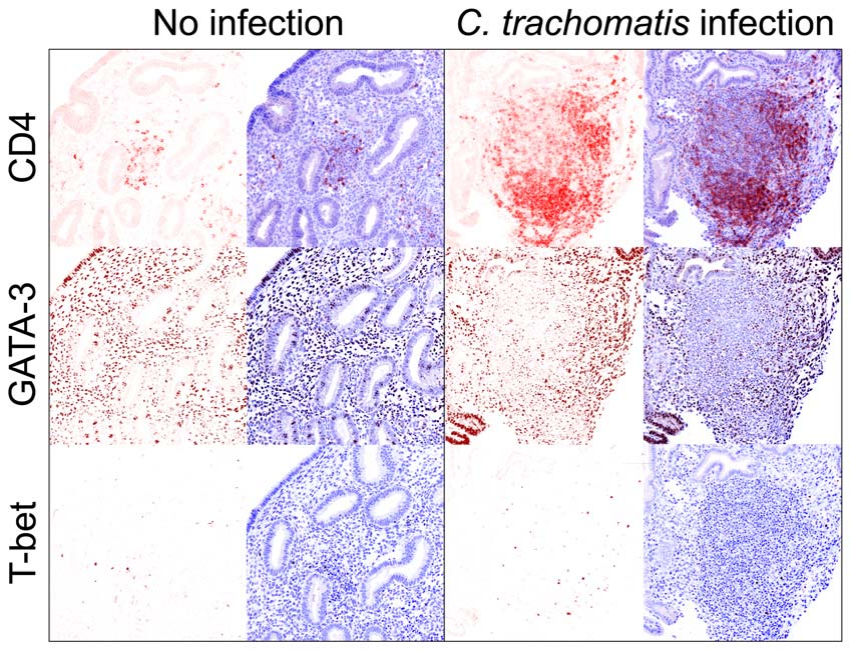
Endometrial *Chlamydia* infection is associated with the presence of CD4^+^ T cell aggregates and high expression of the T_H_2 transcription factor GATA-3. Sequential sections of paraffin-embedded endometria from women with no identified *C. trachomatis*, *N. gonorrhoeae*, or *T. vaginalis* lower or upper genital tract infection (n = 4) or with endometrial *C. trachomatis* infection (n = 6) were used to immunohistochemically evaluate T-bet or GATA-3 expression (both DAB), and the presence of CD4^+^ mononuclear cells (Vector Red) as described in Methods section. Aggregates of GATA-3^+^ (but not T-bet^+^) and CD4^+^ mononuclear cells were seen in endometrial stroma of *Chlamydia*-infected tissue (representative micrographs shown at X200 magnification). Moreover, only a few CD4^+^ mononuclear cells were present in uninfected endometrial tissue even tough GATA-3 was expressed at high levels in both instances. Right panels show images displaying DAB or Vector Red staining and hematoxylin as counterstain, while left panels show DAB or Vector Red layer alone.

**Figure 5:**
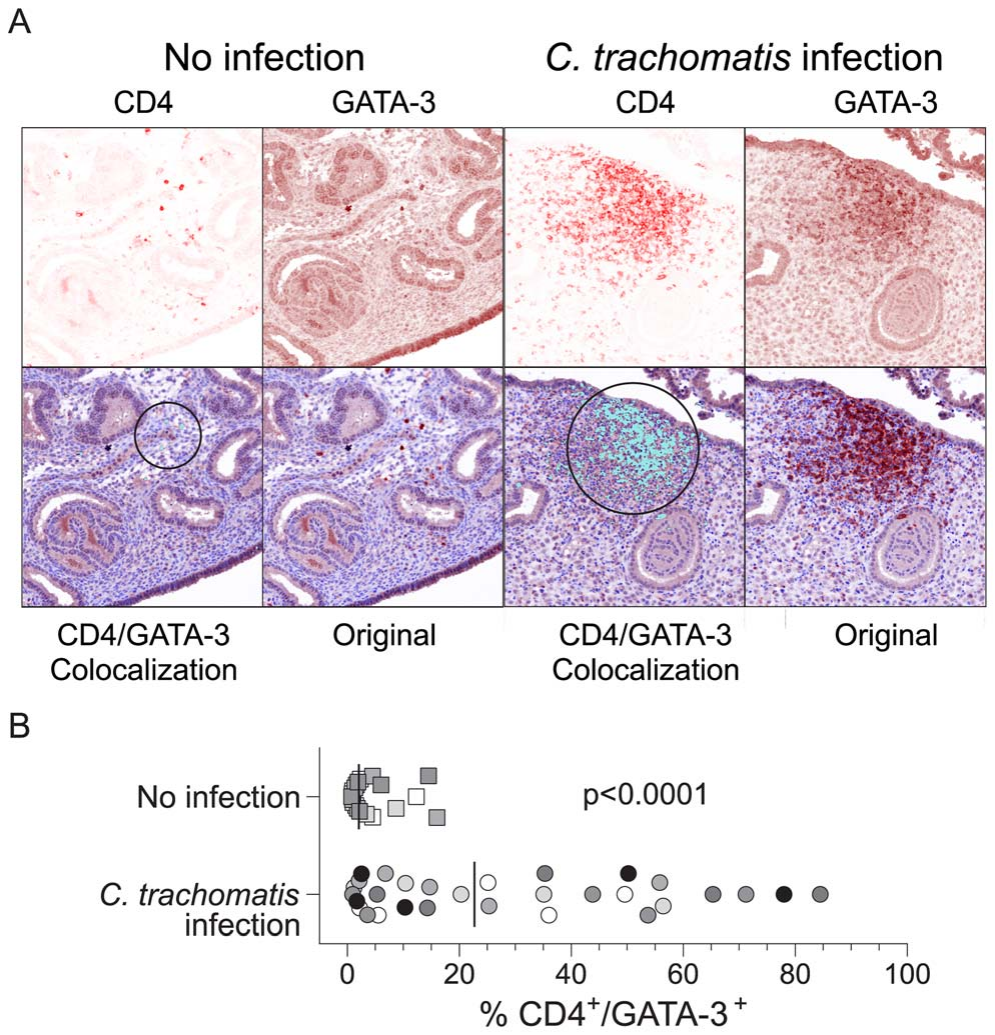
Endometrial *Chlamydia* infection causes infiltration of CD4^+^ T cells expressing GATA-3. Sections of paraffin-embedded endometria from women with no identified *C. trachomatis*, *N. gonorrhoeae*, or *T. vaginalis* lower or upper genital tract infection (n = 4) or with endometrial *C. trachomatis* infection (n = 6) were utilized to simultaneously detect the expression of GATA-3 (DAB) and CD4 (Vector Red) using immunohistochemistry, as described in Methods section. (A) In uninfected endometrial tissue, we observed scarce numbers of CD4^+^ cells coexpressing GATA-3, however, in endometrial tissue from *Chlamydia*-infected women the presence of aggregates of GATA-3^+^ CD4^+^ mononuclear cells was patent (representative micrographs shown at X200 magnification). Upper left panels show images displaying Vector Red staining, while upper right panels show images displaying DAB staining as defined by spectral analysis. Lower right panels show original images used in analysis, and lower left panels show images in which GATA-3 and CD4 colocalization areas have been digitally highlighted (light blue). Circles delineate areas of highest colocalization in images shown. (B) Colocalization of CD4^+^ areas within GATA-3^+^ areas increases dramatically with *Chlamydia* infection, indicating that endometrial *Chlamydia* infection drives the infiltration of GATA-3^+^CD4^+^ T cells that form aggregates. Each symbol represents the percentage of colocalization observed in a single field. Matching colors indicate all the fields evaluated from one specimen. Comparison was performed using a two-tailed Mann-Whitney test (horizontal bars indicate medians).

Based on the substantial T_H_2 response elicited in EB-stimulated peripheral T cells, we further posited that CD4^+^ cells in *Chlamydia*-infected tissue are polarized towards a T_H_2 profile. To test this hypothesis, IHC was used to examine CD4^+^ cell expression of T-bet and GATA-3 (transcription factors regulating T_H_1 and T_H_2 differentiation, respectively) in paraffin-embedded endometrial biopsy sections from women without current *Chlamydia*, *Neisseria gonorrhoeae*, or *Trichomonas vaginalis* infection and women with extant upper genital tract *Chlamydia* infection. As predicted by our ICS assay results, each *Chlamydia*-infected tissue section demonstrated greater expression of GATA-3 than T-bet (representative results shown in Figure 4). Interestingly, expression of GATA-3, but not T-bet, was present in uninfected tissue, indicative of the role this transcription factor plays in estrogen receptor-responsive tissue [22]. Conversely, inspection of five high-powered (X200) fields per specimen revealed GATA-3^+^ CD4^+^ cell numbers were significantly higher in *Chlamydia*-infected vs. uninfected tissue (Figure 5). Taken together, these IHC findings were consistent with preferential secretion of IL-4 by EB-stimulated peripheral T cells from women with extant *Chlamydia* infection (Figure 3).

**Figure 6:**
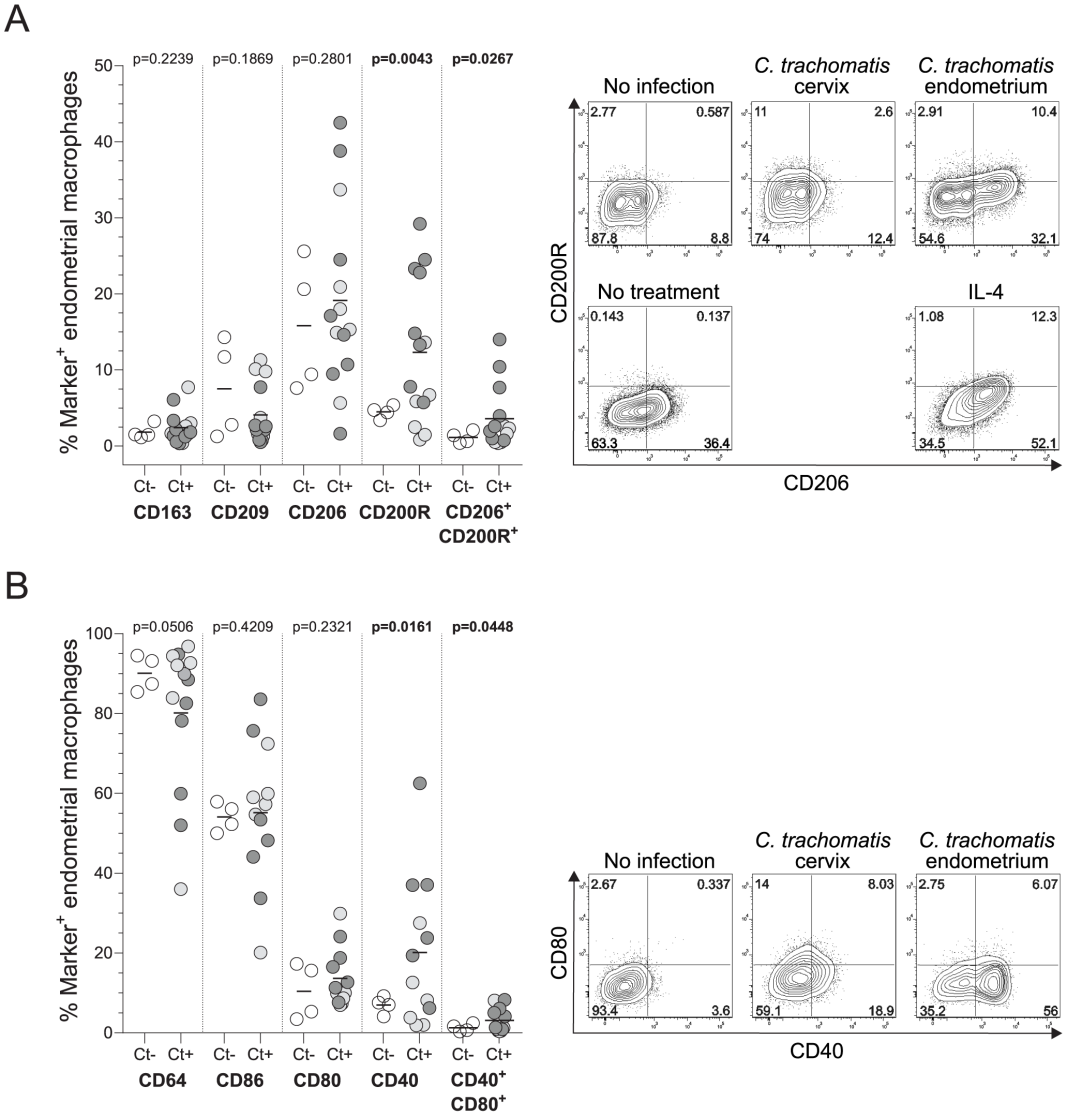
Endometrial *Chlamydia* infection promotes alternative activation of macrophages. Endometrial tissue from women with no identified *C. trachomatis*, *N. gonorrhoeae*, or *T. vaginalis* lower or upper genital tract infection (n = 4), or from women with endocervical or endometrial *C. trachomatis* infection (n = 14 for Panel A; n = 12 for Panel B) were processed for flow cytometric analysis as described in Methods section. Macrophages were identified as FSC-A^int^SSC-A^int^CD45^+^CD15^-^CD14^+^HLA-DR^+^ live cells (as depicted in Figure S3), and 2 monoclonal antibody panels were used to interrogate macrophage differentiation and activation. Panel (A) evaluated expression of CD163, CD209, CD200R and CD206, while panel (B) evaluated expression of CD64, CD80, CD40 and CD86. Comparisons were done using unpaired one-tailed Student t-tests with Welch’s correction (horizontal bars indicate mean values for each group and significant p values are indicated in bold characters). Open circles indicate samples from uninfected controls; light gray circles indicate samples from women with cervical *Chlamydia* infection; and dark gray circles indicate samples from women with endometrial *Chlamydia* infection. Representative contour plots of CD200R, CD206, CD40 and CD80 expression by endometrial macrophages are displayed next to figures. For CD200R and CD206 expression evaluation (A), representative flow plots from peripheral blood monocytes treated with IL-4 (100 U/ml) for 24 hours and the corresponding untreated control are also shown.

Prompted by these results, we returned to our microarray data to examine endometrial transcription factor expression. Based on the high levels of GATA-3 levels expressed in uninfected and *Chlamydia*-infected endometria (Figure 4 and Figure 5), it was not surprising that *Chlamydia* infection induced no significant fold-change in GATA-3 expression. On the other hand, expression of several macrophage-associated transcription factors was significantly modulated by *Chlamydia* infection (Tables 3 and 4). This included increased expression of peroxisome proliferator-activated receptor gamma (PPARG), which promotes polarization of macrophages to the M2 phenotype [23]. As T_H_2 immunity stimulates macrophages that promote fibrosis, tissue remodeling, and wound repair (alternative macrophage activation) [24], [25], we hypothesized that macrophages in *Chlamydia*-infected endometrial tissue display evidence of alternative activation. As predicted, flow cytometry studies showed macrophages in endometria with extant *Chlamydia* infection significantly increased their expression of the CD200R, a marker of alternative macrophage activation and a negative regulator of classical macrophage activation (Figure 6) [26]. Because CD200R binding triggers macrophages to dampen inflammation and suppress collateral damage to host tissue during chronic microbial infection [27]-[29], increased expression of CD200R by macrophages in *Chlamydia*-infected tissue is consistent with the clinical presentation of an infection that persists in genital tract epithelial cells without eliciting overt inflammatory changes. Furthermore, we found that *Chlamydia* infection increased the percentage of endometrial macrophages co-expressing CD200R and CD206 (mannose receptor), another classic marker of alternative macrophage activation (Figure 6) [30]. In addition, *Chlamydia* infection promoted increased macrophage expression of CD40, a costimulatory molecule critical for induction of B cell responses in mucosal tissue [31]. This result correlated with our microarray findings showing *Chlamydia*-infected endometrial tissue had significant enrichment of the B cell development pathway (Table 1) and significantly increased expression of Pax5, a transcription factor essential for commitment to the B lymphocyte lineage [32], [33] (Table 4).

**Table 3:** Transcription factors identified by Ingenuity Pathway Analysis as modulated by endometrial *C. trachomatis* infection (determined by downstream target pools). *

Transcription factor	Fold modulation	-log (p), i.e. 2 ≡ p < 0.01	# Genesmodulated
CEBPA	3.35	6.40	10
ESR1	–2.60	2.23	10
FHL2	–2.18	2.24	5
LEF1	–4.62	1.81	8
NFATC1	2.30	3.52	10
NPAT	5.98	1.71	2
NRIP1	–2.67	1.63	9
PAX8	2.27	1.50	5
PGR	–3.78	1.44	10
RUNX1	3.31	1.40	9
RUNX2	2.04	2.83	10
RUNX3	2.52	5.15	10
SMARCA2	–2.40	1.69	8
TCF3	–2.08	1.57	10
TCF7	2.74	1.71	7
TEAD1	–2.32	1.42	4
TP63	6.38	3.92	10
VDR	4.14	1.58	10

* Ingenuity Pathway Analysis identified 18 known transcription factors that were modulated by endometrial *C. trachomatis* infection whose known downstream targets were significantly enriched among modulated genes. Above table lists those transcription factors, representing 147 occurrences of 96 target genes.

**Table 4:** Transcription factors identified by Ingenuity Pathway Analysis as modulated by endometrial *C. trachomatis* infection (determined by z-score). *

Transcription factor	Activation z-score (must be > 2)	-log (p), i.e. 2 ≡ p < 0.01	Changes consistent
NFκB (complex)	5.98	6.69	49 of 72
SP1	3.42	6.49	25 of 65
CEBPA	3.30	6.40	33 of 54
AHR^a^	2.13	5.13	25 of 41
NCOA1	2.53	5.00	10 of 16
ETS1	3.40	4.37	14 of 29
SPI1	2.50	4.01	12 of 25
TP63	3.35	3.92	19 of 34
STAT1	3.13	3.75	20 of 30
JUN	2.15	3.68	18 of 44
HIF1A	2.58	3.58	22 of 38
SPDEF	2.71	3.50	10 of 14
TP53	2.18	3.00	51 of 103
RELA	2.42	2.98	17 of 37
PPARG	2.17	2.75	21 of 40
FOS	3.00	2.73	20 of 53
CREBBP	2.08	2.66	15 of 25
PAX5	2.17	2.47	5 of 7
RELB	2.19	2.34	7 of 10
EPAS1	2.65	2.28	13 of 21

* Ingenuity Pathway Analysis identified changes in transcription factor activity in the absence of altered transcription factor expression by detecting significantly enriched downstream targets and then confirming that the direction of expression change for each target was in agreement with the known effect (z-score).

a In addition to the transcription factors discussed in the body of text, *Chlamydia* infection was associated with increased expression of the aryl hydrocarbon receptor, a molecule induced by IL-4 in human B cells [37].

In conclusion, the picture of the host response to *Chlamydia* infection of the human female genital tract emerging from our lab is a response skewed towards Type 2 immunity, including differentiation of IL-4-secreting CD3^+^ CD4^+^ and CD3^+^ CD4^-^ cells and stimulation of alternative macrophage activation. Clearly, further interrogation of the phenotype and function of these CD3^+^ CD4^+^ and CD3^+^ CD4^-^ cells is needed, and is an area of active research in our lab. On the other hand, as *Chlamydia* host defense in humans is still thought dominated by highly inflammatory Type 1 immunity [7], [34], our findings already communicate that development of a safe and effective *C. trachomatis* vaccine will require new understanding of immune responses elicited by natural infection and *Chlamydia*-specific immune responses that protect against infection and immunopathological tissue damage. Our study was responsive to the first requisite, offering fresh information about host responses elicited against this obligate intracellular bacterium in the human female genital tract. Regarding the second requisite, our recent [10] and current work implies that Type 2 immunity was evolutionarily selected to control genital *C. trachomatis* infection and minimize immunopathological damage to vital reproductive anatomy. Our work also supports prior observation that IL-13 production by PBMC stimulated with chlamydial antigen correlated with enhanced resistance to *Chlamydia* genital tract re-infection in women [35]. However, only additional work will resolve if *Chlamydia*-specific Type 2 immunity is sterilizing or if Type 2 immunity plays a role in host defense against other intracellular bacterial pathogens.

## Methods

### Ethics Statement

The University of Pittsburgh’s Institutional Review Board approved our study design and procedures (PRO0611062) (PRO09070184) (PRO10010159), and written informed consent was obtained from individuals prior to their participation. While minors/children were eligible for enrollment, none were enrolled and none were assented/consented for enrollment.

### Participants and procedures

Nonpregnant women 15–35 years old at high risk for genital tract infection were eligible for enrollment, while women presenting with symptoms of pelvic inflammatory disease were not. In a separate study, nonpregnant women 18–40 years old that denied history of *Chlamydia* infection were also prospectively enrolled. After participants signed written informed consent, at least 40 ml of peripheral venous blood was collected into sodium heparin-containing blood tubes (Becton-Dickinson). Peripheral blood that was collected from 7 women (average age  =  24.6 years) enrolled with no history of *Chlamydia* infection and 14 women (average age  =  20.8 years) enrolled with existing *Chlamydia* infection (and also collected 1 and 4 months after treatment of infection with 0.25 g ceftriaxone IM and 1 g azithromycin) was used to isolate PBMC by density gradient centrifugation, and these cells were stored in liquid nitrogen prior to their use in ICS assays measuring the effector function of cells that proliferated in response to chlamydial antigen [10], [36]. Cervical swab and endometrial biopsy specimens were used to identify *C. trachomatis* and *N. gonorrhoeae* infection by nucleic acid amplification testing (NAAT), and vaginal swabs were obtained for *T. vaginalis* detection also by NAAT. In women that returned for follow-up visits, absence of these 3 genital tract infections was confirmed with similar testing. Oligonucleotide-based genome array studies utilized endometrial biopsy specimens from 10 women with no current infection and 12 women with existing endometrial *Chlamydia* infection (and without extant *Neisseria* or *Trichomonas* infection as identified by NAAT). Endometrial tissue from 4 women without existing NAAT-detected genital infection and 14 women with current *Chlamydia* infection was used to assess macrophage phenotype by flow cytometry, while paraffin-embedded endometrial tissue from 4 women without existing genital infection and 6 women with current endometrial *Chlamydia* infection was used to evaluate T cell expression of T-bet and GATA-3 by immunohistochemistry (IHC).

### Microarray studies

Endometrial tissue from 10 women with no identified genital infection and 12 women with existing *C. trachomatis* endometrial infection (but no other identified infection) was dissociated into single-cell suspension or placed into RNAlater (Qiagen). Samples underwent total RNA purification using the Qiagen RNeasy Mini Kit in accordance with manufacturer’s instructions and were suspended in nuclease-free water. Inclusion in ensuing *in vitro* amplification assays required a spectrophotometric 260/280 absorption ratio > 1.8 as determined using a NanoDrop spectrophotometer (Thermo Scientific). RIN (RNA Integrity Index) values were determined via electrophoretic analysis (Agilent Bioanalyzer 2100, Agilent Technologies) (results ranged between 5–8). Amplifications were performed with 100 ng total RNA using the NuGEN whole transcription approach involving use of the Ovation FFPE WTA assay (NuGEN) that employed random 3’ primers to eliminate amplification bias. Confirmation of cDNA diversity for each amplification reaction was obtained using the Bioanalyzer 2100 to generate an electrophoretogram regarding sample yield, integrity, and size diversity against a laboratory human RNA standard and a Universal Human Reference RNA (Stratagene). 5 µg of purified cDNA was incubated with fragmentation buffer (NuGEN) for 30 m at 37°C, then 2 m at 95°C. Each cDNA sample underwent hybridization on Affymetrix GeneChip HG U133A 2.0 arrays that contained transcripts representing the functionally characterized human genome. In summary of this process, fragmented cDNA was combined with water in hybridization cocktails to a final volume of 220 µl, and 130 µl of this cocktail was hybridized on each array for 18 h at 45°C. Arrays were washed, stained with streptavidin-phycoerythrin in a GeneChip Fluidics Station 450 (Affymetrix), and scanned using a GeneChip Scanner 3000 (Affymetrix). Quality control parameters were derived from the MAS 5.0 algorithm of the Expression Console software (v. 1.2.0.20; Affymetrix), and expression data derived from raw intensity files generated by this algorithm. Of 22,277 chip panels (i.e., transcript sequences) gauged, 7,759 panels showed ≥ 2-fold change in average gene expression between infected and uninfected tissue. Among such panels, we required the higher expressing group to show detectable transcript (i.e., a “Present” call) in at least 2/3 of samples (i.e., 7 of 10 for uninfected controls and 8 of 12 for infected women). Dunn’s test was then used to determine significance of the differences between the two groups. Selecting differences between mean ranks greater than 5.45 (α  =  0.05) identified 1329 panels, representing 1087 unique characterized genes which have Gene Symbols listed at the http://www.ncbi.nlm.nih.gov/gene website. These 1329 panels were submitted to the Ingenuity Pathways Analysis website which parsed data into 36 significantly enriched canonical pathways consisting of 509 occurrences of 206 unique, characterized genes. Microarray data was deposited to Gene Expression Omnibus (GEO) repository under accession number GSE41075, following MIAME (Minimum Information About a Microarray Experiment) guidelines.

### Flow cytometry studies

For ICS assays, *C. trachomatis* serovar D elementary bodies (EB) were inactivated by γ-irradiation (lack of infectivity confirmed by an absence of inclusion forming units (IFU) when EB doses equivalent to 10^7^ IFU were inoculated onto HeLa cell monolayers and incubated 48 h at 37°C/5% CO_2_). As described elsewhere, PBMC labeled with CellTrace™ Violet cell proliferation dye (Invitrogen) were stimulated with inactivated EB to allow simultaneous quantification of IFN-γ, TNF, IL-4, and IL-17 production by T cells that proliferated in response to chlamydial antigen [36]. Isotype controls were included to establish gates that determined intracellular cytokines production by live CD3^+^ CD4^+^ or CD3^+^ CD4^-^ cells. Stimulation indices were calculated as the quotient of (% CD3^+^CD4^+^ or CD3^+^ CD4^-^ cells proliferating in cultures that received EB) and (% CD3^+^CD4^+^ or CD3^+^CD4^-^ cells proliferating in unstimulated cultures). An adjusted percentage of proliferating, cytokine-producing CD3^+^CD4^+^ or CD3^+^CD4^-^ cells was calculated as the difference between [(% CD3^+^CD4^+^ or CD3^+^CD4^-^ cells proliferating in cultures that received EB) (% cytokine-producing CD3^+^CD4^+^ or CD3^+^CD4^-^ cells proliferating in cultures that received EB)] and [(% CD3^+^CD4^+^ or CD3^+^CD4^-^ cells proliferating in unstimulated cultures) (% cytokine-producing CD3^+^CD4^+^ or CD3^+^CD4^-^ cells proliferating in unstimulated cultures)]. Normality of the data was determined using the D’Agostino–Pearson omnibus test, and statistical tests chosen based on data distribution and the number of comparisons made (p values < 0.05 were considered significant). As applicable, T cell proliferation was compared with 1-tailed Wilcoxon matched-pair signed rank tests or 1-way ANOVA and Dunnett’s method for multiple comparisons. Intracellular cytokine levels were compared with Friedman or Kruskal-Wallis tests and, as indicated, Dunn’s post-hoc test. For macrophage phenotype assays, cryopreserved endometrial cells were thawed and processed at ice-cold temperatures. Single-cell suspensions were stained with LIVEâDEAD® fixable aqua dead cell stain (Invitrogen), and incubated with various combinations of the following optimally titrated monoclonal antibodies: FITC-conjugated anti-HLA-DR FITC (G46-6), PE-conjugated anti-CD163 (HGI/61), PE-Cy7-conjugated anti-CD80 (L307.2), PerCP-Cy5.5-conjugated anti-CD45 (2D1), APC-conjugated anti-CD40 (5C3), V500-conjugated anti-CD15 (HI98) (all BD Biosciences); PE-Cy7-conjugated anti-CD209 (eB-h209), APC-eF780-conjugated anti-CD14 (61D3), eF450-conjugated anti-CD206 (19.2) (all eBioscience); PE-conjugated anti-CD64 (10.1), BV421-conjugated anti-CD86 (IT2.2) (all BioLegend); and AF647-conjugated anti-CD200R (OX108) (AbD Serotec). Cells were washed and fixed in BD Cytofix™ Fixation Buffer (BD Biosciences). Relative expression of the different markers in macrophages present in endometrial tissue from uninfected or women with upper or lower genital tract *Chlamydia* infection was compared using the unpaired, one-tailed Student t-tests with Welch’s correction. In flow cytometry studies, cells were collected on a LSR II cytometer (BD Biosciences), and evaluated using FACSDiva (BD Biosciences) and FlowJo (Tree Star) software. Statistical analyses were performed using Prism® 6 software (GraphPad), and figure legends specify the particular statistical analysis performed.

For ICS assays, *C. trachomatis* serovar D elementary bodies (EB) were inactivated by γ-irradiation (lack of infectivity confirmed by an absence of inclusion forming units (IFU) when EB doses equivalent to 10^7^ IFU were inoculated onto HeLa cell monolayers and incubated 48 h at 37°C/5% CO_2_). As described elsewhere, PBMC labeled with CellTrace™ Violet cell proliferation dye (Invitrogen) were stimulated with inactivated EB to allow simultaneous quantification of IFN-γ, TNF, IL-4, and IL-17 production by T cells that proliferated in response to chlamydial antigen [36]. Isotype controls were included to establish gates that determined intracellular cytokines production by live CD3^+^ CD4^+^ or CD3^+^ CD4^-^ cells. Stimulation indices were calculated as the quotient of (% CD3^+^CD4^+^ or CD3^+^ CD4^-^ cells proliferating in cultures that received EB) and (% CD3^+^CD4^+^ or CD3^+^CD4^-^ cells proliferating in unstimulated cultures). An adjusted percentage of proliferating, cytokine-producing CD3^+^CD4^+^ or CD3^+^CD4^-^ cells was calculated as the difference between [(% CD3^+^CD4^+^ or CD3^+^CD4^-^ cells proliferating in cultures that received EB) (% cytokine-producing CD3^+^CD4^+^ or CD3^+^CD4^-^ cells proliferating in cultures that received EB)] and [(% CD3^+^CD4^+^ or CD3^+^CD4^-^ cells proliferating in unstimulated cultures) (% cytokine-producing CD3^+^CD4^+^ or CD3^+^CD4^-^ cells proliferating in unstimulated cultures)]. Normality of the data was determined using the D’Agostino–Pearson omnibus test, and statistical tests chosen based on data distribution and the number of comparisons made (p values < 0.05 were considered significant). As applicable, T cell proliferation was compared with 1-tailed Wilcoxon matched-pair signed rank tests or 1-way ANOVA and Dunnett’s method for multiple comparisons. Intracellular cytokine levels were compared with Friedman or Kruskal-Wallis tests and, as indicated, Dunn’s post-hoc test. For macrophage phenotype assays, cryopreserved endometrial cells were thawed and processed at ice-cold temperatures. Single-cell suspensions were stained with LIVEâDEAD® fixable aqua dead cell stain (Invitrogen), and incubated with various combinations of the following optimally titrated monoclonal antibodies: FITC-conjugated anti-HLA-DR FITC (G46-6), PE-conjugated anti-CD163 (HGI/61), PE-Cy7-conjugated anti-CD80 (L307.2), PerCP-Cy5.5-conjugated anti-CD45 (2D1), APC-conjugated anti-CD40 (5C3), V500-conjugated anti-CD15 (HI98) (all BD Biosciences); PE-Cy7-conjugated anti-CD209 (eB-h209), APC-eF780-conjugated anti-CD14 (61D3), eF450-conjugated anti-CD206 (19.2) (all eBioscience); PE-conjugated anti-CD64 (10.1), BV421-conjugated anti-CD86 (IT2.2) (all BioLegend); and AF647-conjugated anti-CD200R (OX108) (AbD Serotec). Cells were washed and fixed in BD Cytofix™ Fixation Buffer (BD Biosciences). Relative expression of the different markers in macrophages present in endometrial tissue from uninfected or women with upper or lower genital tract *Chlamydia* infection was compared using the unpaired, one-tailed Student t-tests with Welch’s correction. In flow cytometry studies, cells were collected on a LSR II cytometer (BD Biosciences), and evaluated using FACSDiva (BD Biosciences) and FlowJo (Tree Star) software. Statistical analyses were performed using Prism® 6 software (GraphPad), and figure legends specify the particular statistical analysis performed.

## IHC studies

Paraffin-embedded endometrial tissues from uninfected women and women with extant endometrial *Chlamydia* infection (but no other identified genital tract infection) were stained with polyclonal antibodies detecting GATA-3 or T-bet (both Abcam) and/or a monoclonal antibody detecting CD4 (Dako). This was followed by signal detection that used brown 3,3’ diamino benzidine (DAB) (Dako) and Vector Red (Vector), respectively. For subsequent evaluation, conventional bright field images were acquired using a Cri Nuance spectral analyzer (CRi), and resultant images used to reconstruct multiple spectral distributions and define the intensity and overlap of DAB and Vector Red staining per pixel using CRi Nuance software. Staining intensities were then converted to composite false color images. Finally, to determine relative frequency of CD4^+^ areas overlapping GATA-3^+^ areas five random fields (X200) that contained intact tissue were analyzed per specimen.

## Supporting Information

Figure S1 **Peripheral T cells from women with existing or treated** ***Chlamydia*** **infection proliferated in response to stimulation with C. trachomatis elementary bodies (EB).** Peripheral blood mononuclear cells (PBMC) isolated from women at enrollment and 1-month and 4-month follow-up visits were cultured 96 h in presence of inactivated EB or media alone for 96 h. (A, B) T cells from women with no history of *Chlamydia* infection (n = 7) did not show increased proliferation in response to chlamydial antigen stimulation. (C, D) Peripheral CD3^+^CD4^+^ and CD3^+^CD4^-^ cells from women with existing or treated *Chlamydia* infection (total n = 42, representing the 3 samples taken at indicated time points from 14 women) significantly increased proliferation in response to EB stimulation. Comparisons were made using one-tailed Wilcoxon matched-pairs signed rank test. Open circles represent results from samples not exposed to chlamydial antigen; gray circles represent samples that were stimulated with inactivated EB.
(PDF)

Figure S2 **IL-4 is the predominant and most persistent cytokine produced by peripheral T cells that proliferated in response to ex vivo stimulation with inactivated EB.** PBMC were cryopreserved from women with an existing endocervical or endometrial *Chlamydia* infection (n = 14) at enrollment and again 1 and 4 months after their initiation of anti-chlamydial antimicrobial therapy. Cells were thawed, cultured 96 h in the presence of inactivated EB, and processed for flow cytometric evaluation of IFN-γ, TNF, IL-4, and IL-17 production as described in Methods section. Total cytokine secretion was determined for CD3^+^CD4^+^ (A) and CD3^+^CD4^-^ (B) cells that proliferated in response to inactivated EB, and comparisons performed using Friedman test and Dunn’s post-hoc test (horizontal bars indicate medians). Grey boxes indicate the pairs considered in the comparison for each indicated p value, and significant p values are indicated in bold characters.
(PDF)

Figure S3 **Gating strategy used to identify macrophages infiltrating endometrial tissue.** Cryopreserved endometrial cells were processed for flow cytometric analysis as described in Methods section. Contour plots depict the gating strategy used to define macrophage populations within endometrial cell suspensions. Plots show in sequence the gating hierarchy used to interrogate for CD45^+^, live non-CD15^+^ cells, singlets, and finally to define the macrophage population as CD14^+^HLA-DR^+^(red gate). Representative contour plots displaying expression of some of the surface markers evaluated are also shown (red overlay indicates CD14^+^HLA-DR^+^ cells).
(TIF)
