# Supplementary figures and images for: Human Female Genital Tract Infection by the Obligate Intracellular Bacterium Chlamydia trachomatis Elicits Robust Type 2 Immunity
Source: PLoS One. 2013 Mar 13;8(3):e58565. doi: 10.1371/journal.pone.0058565 (PMC3603585; doi:10.1371/journal.pone.0058565)

A

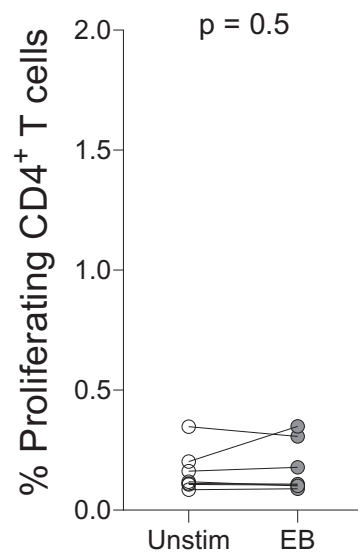

B

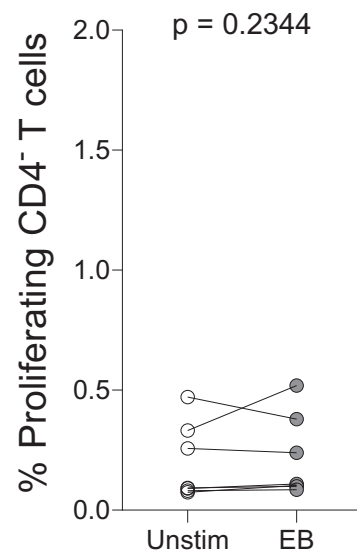

C

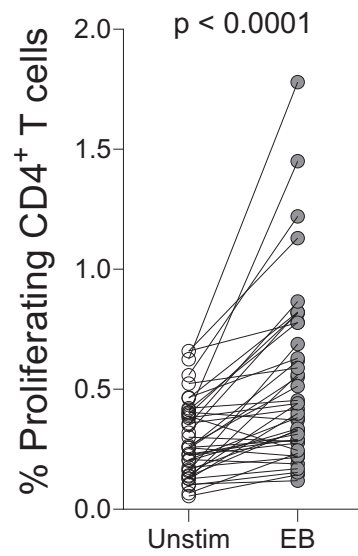

D

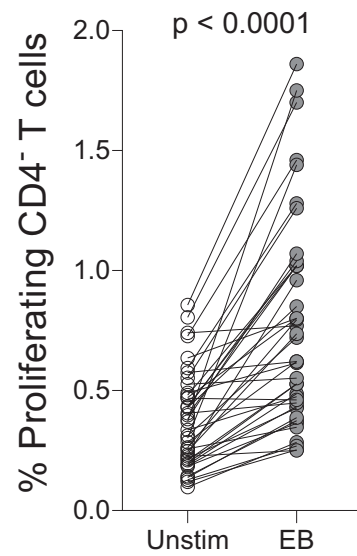

Supplement: Figure S1 — Peripheral T cells from women with existing or treated Chlamydia infection proliferated in response to stimulation with C. trachomatis elementary bodies (EB). Peripheral blood mononuclear cells (PBMC) isolated from women at enrollment and 1-month and 4-month follow-up visits were cultured 96 h in presence of inactivated EB or media alone for 96 h. (A, B) T cells from women with no history of Chlamydia infection (n = 7) did not show increased proliferation in response to chlamydial antigen stimulation. (C, D) Peripheral CD3+CD4+ and CD3+CD4- cells from women with existing or treated Chlamydia infection (total n = 42, representing the 3 samples taken at indicated time points from 14 women) significantly increased proliferation in response to EB stimulation. Comparisons were made using one-tailed Wilcoxon matched-pairs signed rank test. Open circles represent results from samples not exposed to chlamydial antigen; gray circles represent samples that were stimulated with inactivated EB. (PDF) [file pone.0058565.s001.pdf]

A

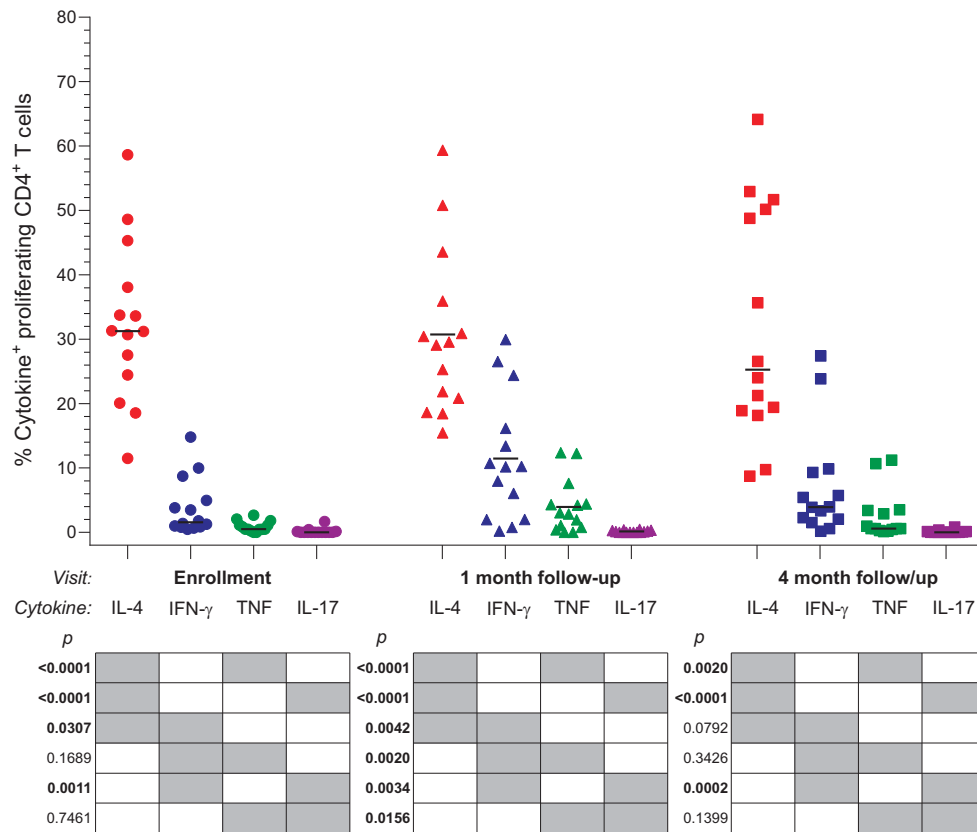

B

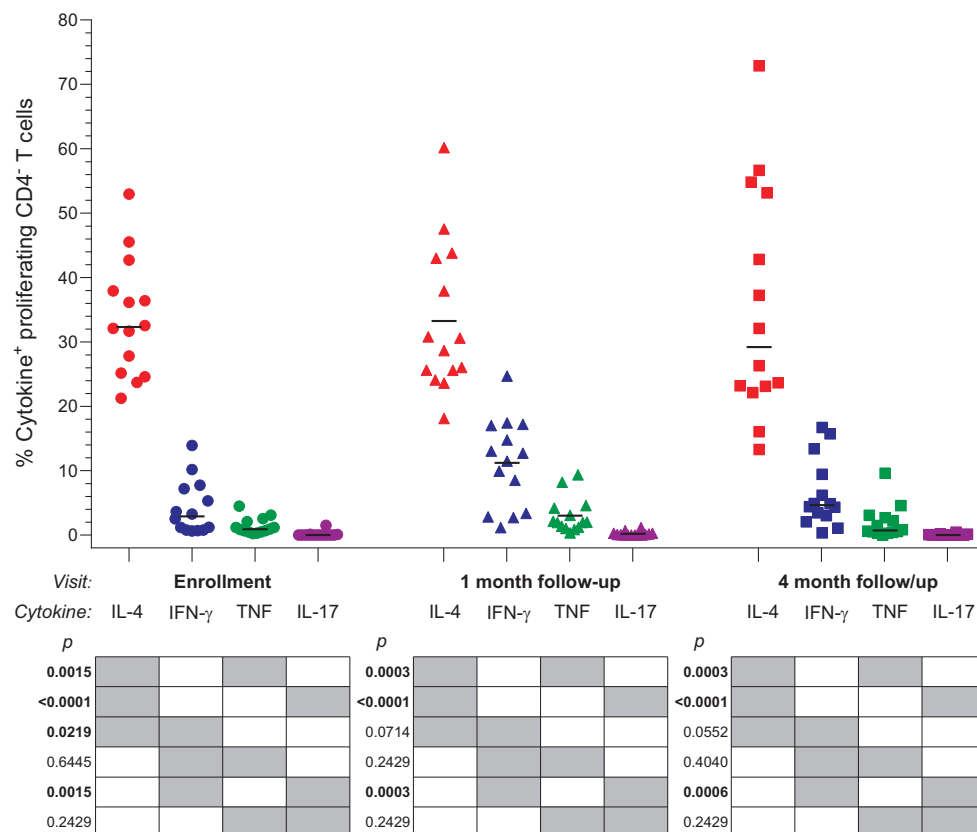

Supplement: Figure S2 — IL-4 is the predominant and most persistent cytokine produced by peripheral T cells that proliferated in response to ex vivo stimulation with inactivated EB. PBMC were cryopreserved from women with an existing endocervical or endometrial Chlamydia infection (n = 14) at enrollment and again 1 and 4 months after their initiation of anti-chlamydial antimicrobial therapy. Cells were thawed, cultured 96 h in the presence of inactivated EB, and processed for flow cytometric evaluation of IFN-γ, TNF, IL-4, and IL-17 production as described in Methods section. Total cytokine secretion was determined for CD3+CD4+ (A) and CD3+CD4- (B) cells that proliferated in response to inactivated EB, and comparisons performed using Friedman test and Dunn’s post-hoc test (horizontal bars indicate medians). Grey boxes indicate the pairs considered in the comparison for each indicated p value, and significant p values are indicated in bold characters. (PDF) [file pone.0058565.s002.pdf]

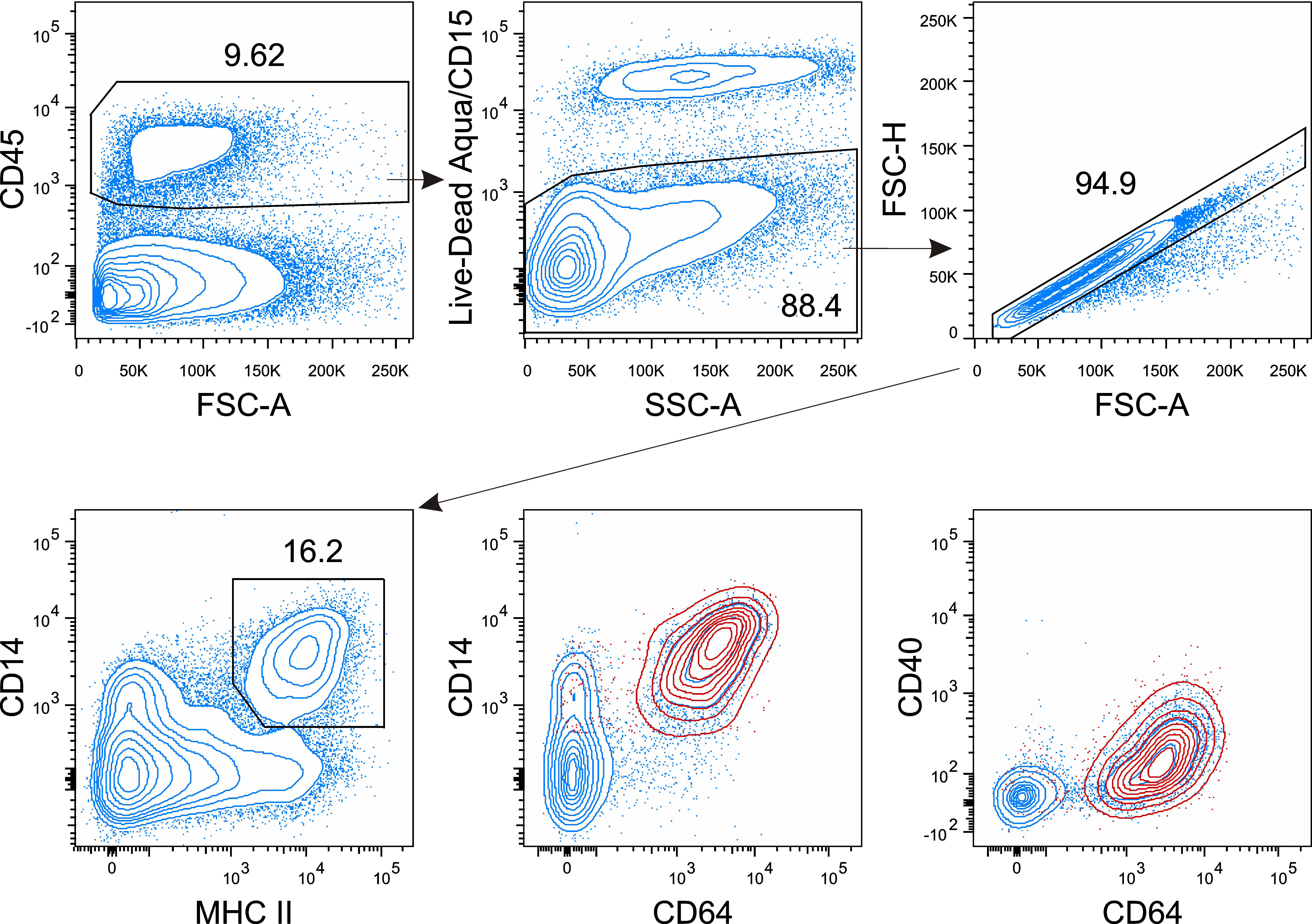

Supplement: Figure S3 — Gating strategy used to identify macrophages infiltrating endometrial tissue. Cryopreserved endometrial cells were processed for flow cytometric analysis as described in Methods section. Contour plots depict the gating strategy used to define macrophage populations within endometrial cell suspensions. Plots show in sequence the gating hierarchy used to interrogate for CD45+, live non-CD15+ cells, singlets, and finally to define the macrophage population as CD14+HLA-DR+(red gate). Representative contour plots displaying expression of some of the surface markers evaluated are also shown (red overlay indicates CD14+HLA-DR+ cells). (TIF) [file pone.0058565.s003.tif]
